# Neurobrucellosis presenting as an intra-medullary spinal cord abscess

**DOI:** 10.1186/1476-0711-4-14

**Published:** 2005-09-16

**Authors:** Girish V Vajramani, Mahantesh B Nagmoti, Chidanand S Patil

**Affiliations:** 1 Department of Neurosurgery, Jawaharlal Nehru Medical College and KLES Hospital, Belgaum, Karnataka, India; 2 Department of Microbiology, Jawaharlal Nehru Medical College and KLES Hospital, Belgaum, Karnataka, India

**Keywords:** Neurobrucellosis, *Brucella melitensis*, spinal cord, intra-medullary abscess, MRI

## Abstract

**Background:**

Of the diverse presentation of neurobrucellosis, intra-medullary spinal cord abscess is extremely rare. Only four other cases have been reported so far. We present a case of spinal cord intra-medullary abscess due to *Brucella melitensis*.

**Case presentation:**

A forty-year-old female presented with progressive weakness of both lower limb with urinary incontinence of 6 months duration. She was febrile. Neurological examination revealed flaccid areflexic paraplegia with T_10 _below sensory impairment including perianal region. An intramedullary mass was diagnosed on Magnetic Resonance Image (MRI) scan extending from T_12 _to L_2_. At surgery, a large abscess was encountered at the conus medullaris, from which *Brucella melitensis *was grown on culture. She was started on streptomycin and doxycycline for 1 month, followed by rifampicin and doxycycline for 1 month. At 2-year follow-up, she had recovered only partially and continued to have impaired bladder function.

**Conclusion:**

Neurobrucellosis, if not treated early, can result in severe neurological morbidity and sequale, which may be irreversible. Hence it is important to consider the possibility of neurobrucellosis in endemic region and treat aggressively.

## Background

The presentation of neurobrucellosis, which encompass neuro-psychiatric disorders in brucellosis, is varied. It can present in acute form as meningoencephalitis or in chronic form, where there is involvement of peripheral nervous system or central nervous system. Chronic form includes epidural granuloma, demyelination of brain or spinal nerve roots, long tract degeneration etc. Meningitis has been the most frequent presentation, occurring in about 50% of the cases [1]. Extensive intra-medullary spinal cord abscess due to *Brucella*, is exceedingly rare. Only four other cases have been reported so far [2-5]. We present a case of intra-medullary spinal cord abscess in which *Brucella melitensis *was cultured from pus.

## Case presentation

**Figure 1:**
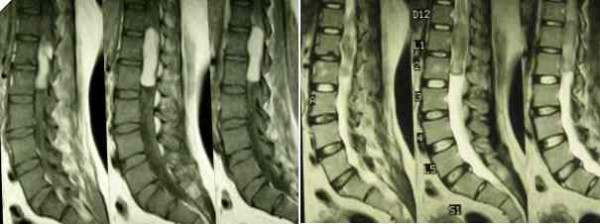
MRI scan showing the intramedullary lesion at conus medullaris. The lesion is hyperintense on T_1_WI and isointense on T_2_WI.

A forty-year-old housewife, from a very low socio-economic status group, presented with history of gradually progressive weakness of left lower limb of 6 months duration, rapidly progressive weakness of right lower limb of 8 days duration and urinary incontinence of 6 months duration. She had been living near a very unhygienic abattoir and admitted to drinking unpasteurised goat's milk. There was history of fever on and off with night sweats. There was no history trauma or past history of tuberculosis. On examination, she was moderately built and nourished. General physical examination was normal. She was febrile with a temperature of 99°F (37.2°C). Vital parameters were normal. Neurologically she was conscious, alert and orientated. Cranial nerve examination was normal. There was no papilledema and meningeal signs were absent. She had flaccid areflexic paraplegia with power 0/5 (MRC grade). She had impaired sensations in both lower limbs with a level at T_10_. Perianal sensations were impaired and she had poor anal tone. Routine haematological parameters revealed a total white blood cell (WBC) count of 13,980/cu mm with neutrophil predominance. Erythrocyte sedimentation rate ESR (Westergreen) was 50 mm in 1 hour. Standard agglutination test (tube) titer was 1:320 and 2-mercaptoethanol agglutination test titer was 1:80. Plain radiograph of lumbosacral spine was normal. MRI scan of the spine showed a lesion in the spinal cord extending from lower part of T_12 _to L_2_. It was hyper-intense on T_1_WI and iso-intense on T_2_WI There was cord edema extending cranially up to T_10 _(figure 1).

**Figure 2:**
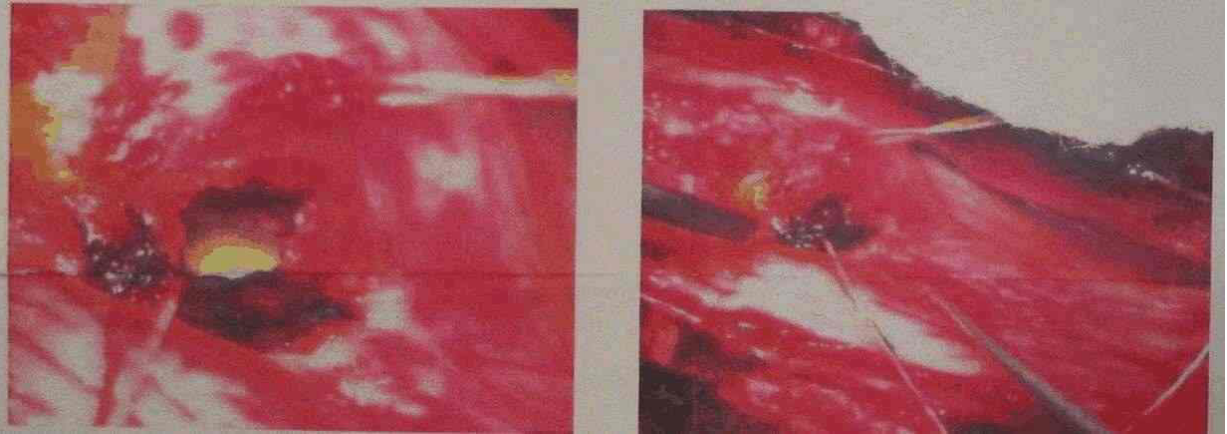
Operative microphotograph showing the intra-medullary abscess.

She underwent T_11 _to L_3 _laminectomy. The lower end of the cord and the conus medullaris were swollen and the cauda equina nerve roots were pushed to the right side. Myelotomy was done at the conus level. At a depth of about 0.5 cm, purulent fluid was encountered, which was sent immediately for microbiological analysis. Under operating microscope the abscess cavity was visualized through the limited myelotomy (fig 2). The abscess was completely evacuated, after which the cord and conus had become lax and pulsating well. Dura was closed completely.

Pus revealed gram-negative bacilli. It was inoculated aerobically [Brucella agar, chocolate and MacConkey media], and anaerobically [Kanamycin-vancomycin laked sheep blood agar (KVLB) and Bacteroides bile esculin agar (BBE)]. Brucella agar and CA were incubated in CO_2 _jar and after 2 days minute translucent colonies were seen. Gram stain from culture showed gram-negative bacilli. Oxidase, catalase, and urease test were positive. There was no H_2_S production and it was resistant to dye inhibition. The organism was confirmed as *Brucella melitensis *[6,7]. The organism isolated in blood culture taken preoperatively, also was identified as *Brucella melitensis*.

Postoperatively she had fever, headaches and vomiting lasting for about 1 week. It subsided once antibiotics were instituted. She was started on injection streptomycin 1 gm once a day for 1 month with oral doxycycline 100 mgm twice a day for 1 month. After one month she received oral rifampicin 450 mgm once a day with oral doxycycline 100 mgm twice a day for 1 month. Dexamethasone was given only perioperatively and was rapidly tapered and stopped in the post-operative period. Post operatively she gradually improved in neurological status. At 2-year follow up she had grade 3/5 power in both lower limbs and was mobilising on a wheel chair. The urinary symptoms did not resolve and she continues to be on Foley catheter. She refused a repeat MRI scan, as she could not afford it.

## Discussion

Brucellosis is a common zoonosis in many parts of the world. Various types of central nervous system (CNS) involvement in brucellosis have been reported [1,8-11], the estimated incidence varying from 5–25% in different series, with an average of 3–5% [1,11,12]. The exact pathogenesis of CNS involvement is not clear. Various mechanisms have been postulated. It is known that *Brucella *organisms are capable of prolonged intracellular survival within phagocytes. Decreased host immunity may allow the organisms to proliferate [13]. The organism may act directly or indirectly through its endotoxins [1]. Immune mediated demyelination has been proposed to explain certain chronic forms of neurobrucellosis [11]. The cord or nerve root may secondarily be involved due to spondylitis, vasculitis and arachnoiditis [10].

Brucellosis is not uncommon in Belgaum district of Karnataka. Various forms of brucellosis including neurobrucellosis, have been reported from this region [8,9,12]. None of them had an intramedullary involvement of spinal cord. There are only four previous reports, in the world literature, of intramedullary involvement by *Brucella *(table 1). Systemic brucellosis was seen in all of them including the present case. Only in the present case was *Brucella melitensis *cultured from the intramedullary pus-in others either, it was not biopsied or there was no growth, the infection being suspected because of presence of systemic brucellosis. In the case reported in 1994 by Cokca et al, a 17-year old boy had an intramedullary dermoid that was infected with *Brucella abortus *type 3. At surgery, multiple cavitary abscesses containing hair was drained. He responded well to surgical drainage and medical treatment [2]. Bingol et al, in 1999, presented a 40-year old female who had a 10 × 30 mm intramedullary granuloma with surrounding oedema on MRI scan. This was presumed to be a *Brucella *granuloma as she was diagnosed and treated for systemic brucellosis about 4 months ago. She needed extended period of antibiotics with which there was complete resolution of the lesion as seen on the follow-up scan [3]. Novati et al, in 2002, described a 24-year old man who was diagnosed to have a focal abscess, 15 mm in diameter, within the dorsal tract of the spinal cord with perilesional oedema. Blood and bone marrow aspirate had grown *Brucella melitensis *and the patient was started on antibiotics for a period of 6 months, following which the abscess resolved [4]. Helvaci et al, in 2002, described a 15-year-old girl with systemic brucellosis, who had a well-circumscribed intramedullary mass at T_11_–T_12_. This was drained and biopsied as she did not respond to antibiotics alone. Histopathology showed non-caseating granuloma with chronic inflammation. Cultures of the purulent material were negative. She was treated with a total of 8 weeks of antibiotics with which she recovered considerably [5].

**Table 1:** Cases of neurobrucellosis with intramedullary spinal cord involvement.

**Case**	**Age/Sex**	**Risk factor**	**Lesion**	**Pus culture**	**Blood culture**	**Serology**	**Treatment**	**Systemic brucellosis**
Cokca et al, 1994	17-year-old boy	Regular consumption of cows milk, living in rural area near breeding animals	Intramedullary dermoid cyst (T11-L2)	*Brucella abortus *biotype 3	*Brucella abortus *biotype 3	not mentioned	Surgical+Medical	present
Bingol et al, 1999.	40-year-old female	Raising sheep and consuming raw milk	Intramedullary granuloma at T5	not done	No growth	positive	Medical	present
Novati et al, 2002.	24-year-old male	Consumption of fresh goats cheese	Intramedullary abscess at T3	not done	*Brucella melitensis*	positive	Medical	present
Helvaci et al, 2002	15-year-old girl	Consumption of cheese made from raw goats milk	Intramedullary abscess at T11-T12	no growth	no growth	positive	Surgical+Medical	present
Present case, 2005	40-year-old female	Living near abattoir, consumption of meat and goat's milk.	Intramedullary abscess at Conus	*Brucella melitensis*	*Brucella melitensis*	positive	Surgical+Medical	present

In the present case, there was direct involvement of spinal cord by the *Brucella melitensis *leading to a chronic progressive neurological impairment. Despite the raised pre-operative titres of antibody to brucella antigen and high prevalence of brucellosis in this region, the possibility of the cord lesion to be of *Brucella *origin was not considered pre-operatively, probably because of the rarity of brucella abscess in the spinal cord. The post-operative fever and headache could have been due to transient meningitis resulting from contamination of the CSF space during surgery. This, fortunately, responded well to the antibiotics.

There are no specific guidelines regarding the antibiotic regimens and duration of treatment for neurobrucellosis. The duration of treatment varies from 8 weeks to 2 year depending upon individual cases, surgical or medical line of treatment and response to the treatment. Drugs such as doxycycline, rifampicin and trimethoprim-sulfamethoxazole have been found effective due to their good CNS penetration and synergistic actions [10,14]. Tetracycline and streptomycin are good for systemic brucellosis, although their CNS penetration is poor. However, as most of these patients have systemic brucellosis as well, they should be covered with these antibiotics, especially in initial stages. In the present case, streptomycin and doxycycline was given for 1 month followed by rifampicin and doxycycline for 1 month. As the abscess was emptied of it contents completely under operating microscope, antibiotics were given for only 2 months in the post-operative period.

## Conclusion

1. It is important to consider the possibility of intra-medullary abscess as a presentation of neurobrucellosis, especially in endemic region.

2. Prompt detection and neurosurgical drainage with antibiotics usually results in resolution of the infection.

3. The duration of antibiotics is variable and depends upon, a. the type of lesion, abscess or granuloma, b. whether surgically drained or not, and c. response to treatment. Steroids should be considered initially, especially if edema is demonstrated on the scan.

4. Increased awareness and early intervention could prevent the neurological disability and improve the functional outcome.

## Competing interests

The author(s) declare that they have no competing interests.

## Authors' contributions

GVV cared for the patient and did the operation. He did the literature search, and was involved in the inception of the paper. MBN was involved in microbiological analysis, literature search and drafting the manuscript. CSP was involved in microbial testing, critical review and supervising the paper.
